# Patterns of collaboration and knowledge generated by an Australian rural research centre over 20 years: a co-authorship network analysis

**DOI:** 10.1186/s12961-023-01029-y

**Published:** 2023-08-30

**Authors:** Jodie Bailie, Petr Matous, Ross Bailie, Megan E. Passey

**Affiliations:** 1 University Centre for Rural Health, The University of Sydney, 61 Uralba Street, Lismore, NSW 2480 Australia; 2 School of Public Health, The University of Sydney, Camperdown, Australia; 3 School of Project Management, Faculty of Engineering, The University of Sydney, Camperdown, Australia

**Keywords:** Collaboration, Rural research, Networks, Research capacity strengthening

## Abstract

**Background:**

People living in rural areas have poorer health than their urban counterparts. Although rural health research centres have been promoted as vehicles for improving rural health by contributing evidence to address rural health disadvantage and building research capacity, their characteristics and evolution are poorly understood. Collaboration is known to have an important positive influence on research outputs and research quality. In this study we examine publication outputs from an Australian rural research centre to evaluate how researchers have engaged in research collaboration over a two-decade period.

**Methods:**

A retrospective longitudinal study of publications in peer-reviewed journals from a rural research centre—University Centre for Rural Health (UCRH) —between January 2002 and December 2021. Organisational co-author networks across four periods (2002–2006; 2007–2011; 2012–2016; 2017–2021) were constructed based on author organisational affiliations and examined using social network analysis methods. Descriptive characteristics included organisation types, study design, region of study focus, thematic research trends, Aboriginal and Torres Strait Islander and female authorship, and journal characteristics.

**Results:**

We identified 577 publications with 130 different UCRH-affiliated authors. Publications and the co-author network increased in number and diversity over each period, with an acceleration and a consolidation of the network in the final period. Over time there was an increase in publications related to Aboriginal and Torres Strait Islander health, coupled with an increase in Aboriginal and Torres Strait Islander authorship and collaborations with Aboriginal and Torres Strait Islander organisations; rise in female senior authorship and publication in quartile 1 journals. About two-thirds of publications make no reference to regional or remote populations.

**Conclusion:**

Collaboration in publications increased, expanded, and consolidated, which coincided with an increase in the number and diversity of both co-authoring organisations and UCRH-affiliated authors in the final period. The findings highlight the value of collaborations (including urban and international) in building and strengthening rural health research capacity. With increased capacity and consolidation of the network it is now imperative that research becomes more focussed on understanding and addressing rural health inequities.

## Background

Approximately 30% of Australians, totalling 7 million people, reside in rural and remote areas [1]. In comparison to their urban counterparts, individuals living in rural areas generally experience poorer health outcomes. They face higher rates of chronic diseases, behavioural risk factors, and mortality, along with lower life expectancy [1]. Despite facing an increased burden of illness, people residing in rural areas often encounter limited access to health care due to various complex and unique challenges, including health workforce shortages, cost and geographic distances [1]. Undertaking research that is of specific relevance to rural populations is seen as part of the solution to addressing rural health disadvantage [2–7]. Over the past 25 years, successive Australian governments have been investing in improving rural health through a variety of initiatives, such as the Rural Health Multidisciplinary Training (RHMT) program that invests in 21 universities to establish a network of rural health teaching and research centres [8–10]. Over time these centres have built rural research capability and capacity [9, 11], with a concomitant increase in the number of research publications on rural health [5, 9, 12–15].

The University Centre for Rural Health, situated in the Northern Rivers region of New South Wales, Australia, is one of these RHMT-funded centres [16]. Established in 2001, it delivers rural health training for health professionals and conducts research relevant to the health needs of rural communities [16]. UCRH staff conduct research, supervise research students and support local clinicians to undertake research. While the RHMT funding allocated for research staffing is limited, UCRH staff have been successful in attracting external research funding. In a recent evaluation of the RHMT program in 2020, the UCRH was identified as a good example of a rural health research centre with *‘a strong research program supported through National Health and Medical Research Council (NHMRC) and Australian Research Council (ARC) grants’* (pg. 121) [9].

Collaboration in its many forms (international, national, multi-sector and intraorganizational) is known to have an important influence on research outputs and research quality [5, 17–19]. Equity is an important factor in effective collaborations [20–22]. In Australia there has been a call to increase both female [23] and Aboriginal and Torres Strait Islander [24, 25] leadership and participation in research to address historical imbalances. An expression of collaboration is co-authorship, when two or more authors co-publish in academic journals. Collaboration benefits research through the nature of teamwork and also increases research impact [26].

The national evaluation of RHMT funding has recommended that rural health research centres can be strengthened by *‘building collaborations and networks with [a] central university, across universities and across jurisdictions to progress rurally focused research of national and international significance.’* [9] (page 269). While rural health research centres have been promoted as vehicles for improving rural health outcomes by providing an evidence base to address rural health disadvantage and building research capacity, their characteristics and evolution are not well understood. Co-authorship network analysis [22, 27–29] offers a method of evaluating the growth and emergence of research networks because publications are well documented and quantify research collaboration [17, 22, 28, 29].

In this paper, we use co-authorship network analysis and bibliometrics to assess how researchers from an Australian rural health research centre—the University Centre for Rural Health—have engaged in research collaborations over a 20-year period, and to draw lessons from this analysis to gain insights into success in rural health research. Specifically, our objectives are, to: (1) Describe the number of publications over time for UCRH-affiliated authors, including publication type and study design; (2) Describe the rurality of research settings; (3) Identify changes in research themes; (4) Identify the level of equity in authorship relative both to Aboriginal and Torres Strait Islander status and to gender; (5) Identify publication metrics related to journal types; (6) Investigate the structure and composition of the research network over time.

## Methods

In 2022, we conducted a retrospective longitudinal study of publications in peer-reviewed journals by UCRH-affiliated authors between 2002 and 2021. We used co-authorship network analysis, as described by Fonseca et al. [28] in their work on health sector co-authorship, to retrieve scientific publication details from collaborating authors, standardise entries for authors and organisations, visualise the network and calculate the metrics. As this study used only previously published articles, and did not involve any human subjects, institutional ethics board review was not necessary.

### Data retrieval

Details of publications in peer-reviewed journals (the ‘publications’) were retrieved from administrative records held by the UCRH and duplicate entries removed. Articles were included if (1) at least one author had a UCRH affiliation listed; (2) it was published in a peer-reviewed journal (including commentaries, research articles, systematic reviews, and letters to the editor); and (3) it was published between January 2002 and December 2021.

Where authors had more than one affiliation listed on the publication, our approach involved two steps. Firstly, we aimed to identify if a UCRH affiliation was explicitly mentioned. If such an affiliation was identified, it was recorded as the primary affiliation for that author. In cases where a UCRH affiliation was not specified, we proceeded to consider the first affiliation listed by the author on the publication.

Over the 20-year period, the UCRH has had several name changes, including as Northern Rivers University Department of Rural Health, North Coast Medical Education Collaboration, and Northern Rivers University. In consultation with MP, we categorised all of these affiliations as being from the UCRH and removed any publication that did not list a UCRH affiliation.

### Data categorisation, standardisation, and cleaning

Here we describe the process for categorisation of included publications, with the categories iteratively refined and defined by JB, RB and MP.

#### Organisations

The affiliations of the authors (as per their citation on publications) were coded into universities and research institutes; health services; government departments; local health districts; peak body, training or advocacy organisations; and other.

The following key points were used in the categorisation of publications:

- The author’s university rather than their specific department and, if named, the research institute rather than the university.
- Where authors identified a rural health department of the university—for example, Department of Rural Health, Monash University—we used this affiliation.
- Where an author’s affiliation was nominated as a public hospital, we used the State Health Department with which these organisations were affiliated.
- Health service—refers to services established primarily to provide health care to individuals and includes organisations such as Aboriginal and Torres Strait Islander community-controlled health services, private general practice, specialist clinics, private and public hospitals, and counselling services.
- Government—refers to departments in government at local, state and national levels.
- Peak body, training, advocacy organisation—refers to organisations that operate independently of government, typically with the purpose of addressing a social or political issue or to support health service delivery and training.

We also identified whether the organisation was international, rurally based and established to support rural issues, or Aboriginal and Torres Strait Islander managed.

#### Research themes

As a starting point, JB and MP categorised all publications under the research themes developed for the UCRH Strategic Plan (2018–2020) [30]. These are: healthy start to life; social and environmental health; mental health and social and emotional wellbeing; alcohol and other drugs; chronic disease and co-morbidity; health ageing; and care for an ageing population. In categorising publications, however, it became apparent that we needed to add further research themes. Through an iterative process JB and MP added the following categories: health workforce and student training; agricultural health; infectious diseases; cancer; methods, quality improvement tools and processes; and ‘other’. Publications were then allocated to a category based on their predominant theme. For example, several publications in later periods used quality improvement techniques to improve different areas of care so, where appropriate, we categorised these according to their predominant area of health care.

Given the national priorities of developing the evidence base to improve health outcomes for Aboriginal and Torres Strait Islander people [31] and people with disability [32, 33], we have included the cross-cutting research themes of Aboriginal and Torres Strait Islander health and disability.

#### Region of study setting

Four categories were developed (1) regional and remote only; (2) major city, regional and remote breakdown; (3) no specific reporting of regional or remote; and (4) international.

#### Aboriginal and Torres Strait Islander and female authorship

We examined first and last authorship by gender and Indigenous status. If a paper had only one author, that author was considered the first author because the first author position is traditionally the author responsible for the conceptualisation and writing of the manuscript. Additionally, we considered the last author, as it is commonly understood in health research that the last author is the most senior member on the research team and has provided academic guidance and oversight [34]. In cases where an organisation was listed as the last author, we substituted it with the last individual author mentioned instead of the organisation. Where there was uncertainty in allocating authors to these categories, JB checked with MP and RB and, when necessary, with the corresponding authors themselves. Data were entered into an Excel spreadsheet, and then standardised and cleaned by JB and PM.

#### Publication metrics

We utilised Scimago Journal and Country Rank (www.scimagojr.com) as a tool to assess the quality of journals. When Scimago designates a journal as a Quartile 1 journal, it signifies that the journal ranks in the top 25% of journals in at least one discipline. This ranking is based on various indicators and metrics employed by Scimago, which includes citation counts and journal impact factors. Being in the Quartile 1 category indicates that the journal is among the highest-ranked journals within its specific field or discipline for the year it was published. This ranking signifies that the journal is of high quality and has a strong impact within its academic domain and therefor more likely to contribute to the advancement of knowledge, inform practice and influence policy.

### Network assembly, visualisation and analysis

The evolution of the publications of the research centre was analysed over four periods, with the analysis split into two parts: (1) the network analysis of co-authorship between organisations (i.e. not between individuals); and (2) a descriptive analysis of publications by study design, thematic research trends, author order, rurality of study focus, and Aboriginal and Torres Strait Islander and female authorship.

We first created a node list containing every organisation that has co-authored with the UCRH along with their attributes (unique identifier, organisation name, type and years published), and an edge list representing all pairs of organisations that have been listed as having affiliations with co-authors on the same publication. A single, undirected edge of weight = 1 was assigned for each organisation pair that shared at least one publication in each of the network’s periods. (Co-authorships between members of the same organisation, i.e. self-loops in the network, were not a subject of the present analysis.) No additional weight was given to the number of publications or authors involved or any other attribute. This approach was chosen so that results of the analysis could be directly interpreted in the context of interorganisational collaboration.

Networks were analysed discretely across the four periods, with several measures (defined in Table 1) used to understand the resulting networks. The UCRH has been removed from the presented networks because it is, by definition, connected with everyone in the sample and its inclusion would obscure the underlying network structure. The analysis was carried out in R [35] and package igraph [36].

**Table 1:** Definition of social network terms and their meaning in this study

Measure	Definition and meaning in this study
Node	The node is the basic element of the network being connected. In this study, nodes represent organisations that were affiliated with authors who co-published with the UCRH
Edge or tie	An edge or tie connects two nodes in a network and indicates a relationship between the two. In this study, an edge between two organisations indicates co-authorship of at least one publication
Degree-related measures	Degree-related measures show the number of ties coming from each node and going to each node. A higher co-authorship activity is reflected in higher median and mean degree, and a more connected network overall. Organisations with the degree of zero collaborated exclusively with UCRH without the involvement of any other organisation. Nodes with the maximum degree have the highest number of mutual collaborators in common with UCRH
Degree variance	Degree variance measures the spread of co-authoring activity between high-degree and low-degree nodes. It is also a way to conceptualise centralisation, used especially when dealing with networks of significantly different sizes. High degree variance indicates a presence of centralised hubs in a network to which lower degree nodes connect. It may also be a sign of a high proportion of isolated nodes
Freeman degree centralisation	Freeman degree centralisation quantifies the relative dominance of the highest degree actor in a network. Hub and spoke networks that centre around a single focal point display high Freeman centralisation. The theoretical Freeman degree centralisation maximum would be reached in a hypothetical case of a perfect star diagram in which one of UCRH’s co-authoring organisations was connected to every organisation in the sample through a separate publication, and all the other organisations never co-authored together during the period. In practice, it is relatively easier to get closer to this hypothetical state in smaller networks
Density	Network density is the proportion of the actual number of connections to the theoretically possible maximum number in a network (which is given by the network size defined as the number of its nodes). The possible connections among a set of nodes increases quadratically with the number of nodes. Therefore, density is expected to be lower in large networks with generally similar levels of networking activity expressed by mean degree
Components	Components of a network are network parts that are disconnected from one another. There is a path between all pairs of nodes in the same component and no network path between separate components. The number of disjoint components in a network is a measure of its fragmentation. A fully connected network has only one component that all nodes belong to. An isolated node with no connections is a component of size one
Network diameter	The diameter of the network is the shortest distance between its two most distant nodes. It is measured by the maximum number of edges needed to connect any two nodes in the network
Assortativity	Assortativity is the tendency of nodes being connected to similar nodes in the network. Assortativity values for a certain node characteristic can vary from -1 to + 1, with negative values indicating the prevalence of links between dissimilar nodes, positive values indicating a prevalence of links between similar nodes, and zero in absolute value indicating a non-assortative network [37]. The magnitude of assortativity co-efficients are interpreted in the same way as correlation co-efficients

Definition of terms are informed by Scott, J. Social network analysis: a handbook. Second Edition. London: Sage; 2000 [38]

### Patient and public involvement

No patients or members of the public were involved in the design, analysis or reporting of this study.

## Findings

### Descriptive characteristics of publications

We identified 577 publications, with 130 different UCRH-affiliated authors (Table 2). The number of different UCRH-affiliated authors increased from 12 in period 1 to 83 in period 4 with 11 of these authors each contributing 20 or more publications to the research network. Over time there was an increase in the number and percentage of publications that had a UCRH-affiliated last—or senior—author from 27% (*n* = 8) in period 1 to 40% (*n* = 99) in period 4. First-authored UCRH-affiliated publications increased in number but decreased in percentage from 50% (*n* = 15) in period 1 to 34% (*n* = 84) in period 4.

**Table 2:** Descriptive characteristics of included publications, by periods and total 2002–2021

Indicator	Period 1: 2002–2006	Period 2 2007–2011	Period 3 2012–2016	Period 4 2017–2021	Total 2002–2021
UCRH publications: no of publications	30	113	188	246	577
Distinct UCRH-affiliated authors: no of authors	12	36	50	83	130
UCRH first- and last-authored publications					
UCRH first-authored publications (% of period)	15 (50%)	61 (54%)	74 (39%)	84 (34%)	234 (41%)
UCRH last-authored publications	8 (27%)	39 (35%)	74 (39%)	99 (40%)	220 (38%)
UCRH first- and last-authored publications	6 (20%)	26 (23%)	32 (17%)	45 (18%)	109 (19%)
Publication type					
Research article	24 (80%)	86 (76%)	157 (84%)	200 (81%)	467 (81%)
Letter	0	1 (1%)	6 (3%)	3 (1%)	10 (2%)
Review (systematic, scoping, narrative, etc.)	0	2 (2%)	7 (4%)	13 (5%)	22 (4%)
Commentary/perspective	6 (20%)	23 (27%)	13 (7%)	20 (8%)	62 (11%)
Editorial	0	1 (1%)	2 (1%)	1 (0.4%)	4 (1%)
Study protocol	0	0	3 (2%)	9 (4%)	12 (2%)
Study design					
Qualitative	0	8 (9%)	36 (22%)	58 (26%)	102 (21%)
Quantitative	21 (88%)	65 (75%)	108 (65%)	121 (55%)	315 (64%)
Mixed methods	3 (13%)	14 (16%)	21 (13%)	41 (19%)	79 (16%)
Rurality of research settings					
Regional and remote only	8 (27%)	41 (36%)	52 (28%)	62 (25%)	163 (28%)
Major city, regional and remote breakdown	3 (10%)	4 (4%)	13 (7%)	19 (8%)	39 (7%)
No. specific reporting of regional or remote	19 (63%)	68 (60%)	123 (65%)	165 (67%)	375 (65%)
International setting	0	12 (11%)	12 (6%)	21 (9%)	45 (8%)
Research themes					
Healthy start to life	2 (7%)	20 (18%)	47 (25%)	29 (12%)	98 (17%)
Chronic disease and co-morbidity	0	8 (7%)	42 (22%)	28 (11%)	78 (14%)
Social and environmental health	11 (37%)	8 (7%)	13 (7%)	40 (16%)	72 (12%)
Mental health and social and emotional wellbeing	3 (10%)	12 (11%)	27 (14%)	28 (11%)	70 (12%)
Methods; CQI tools and processes	1 (3%)	9 (8%)	12 (6%)	30 (12%)	54 (9%)
Infectious disease	1 (3%)	2 (2%)	9 (5%)	28 (11%)	40 (7%)
Healthy ageing and care for an ageing population	4 (13%)	12 (11%)	9 (5%)	5 (2%)	30 (5%)
Health workforce/student training	4 (13%)	12 (11%)	9 (5%)	5 (2%)	30 (5%)
Other	2 (7%)	4 (4%)	6 (3%)	8 (3%)	20 (3%)
Cancer	1 (3%)	1 (1%)	0	10 (4%)	12 (2%)
Agricultural health	0	1 (1%)	1 (1%)	3 (1%)	5 (1%)
Cross-cutting research themes					
Aboriginal and Torres Strait Islander health-related topic	0	7 (6%)	39 (21%)	71 (29%)	117 (20%)
Disability-related topic	0 (0%)	0 (0%)	2 (1%)	1 (0%)	3 (1%)
Equity in authorship: Aboriginal and Torres Strait Islander					
At least one Aboriginal and Torres Strait Islander author:	0	2 (2%)	21 (11%)	65 (26%)	88 (15%)
Aboriginal and Torres Strait Islander first author	0	0	0	10 (4%)	10 (2%)
Aboriginal and Torres Strait Islander last author	0	0	2 (1%)	11 (4%)	13 (2%)
Equity in authorship: Female authorship					
Female first author	15 (50%)	80 (71%)	147 (78%)	179 (73%)	421 (73%)
Female last author	0	17 (15%)	58 (31%)	143 (58%)	218 (38%)
Publication characteristics					
Quartile 1 Journal at year of publication	13 (43%)	59 (52%)	121 (64%)	154 (63%)	347 (60%)

(1) Data are presented as number and percentage for each period, therefore percentages will not total to 100% in each row. (2) *CQI* continuous quality improvement, *UCRH* University Centre for Rural Health

Overall, most publications (81%, *n* = 467) were primary research articles, followed by commentaries and perspectives (11%, *n* = 62) (Table 2). Overall, most of the publications used quantitative methods (64%, *n* = 315), and around one fifth employed qualitative methods (21%, *n* = 102) (Table 2).

### Rurality of research settings

As the network evolved there was little change in the percentage of publications that had a regional and remote only focus (between 25 and 36% across the four periods) and about two thirds of publications (between 60 and 67% across the four periods) making no reference to regional or remote populations (Table 2). Overall, 8% (n = 45) of publications had an international focus.

### Research themes

**Fig. 1:**
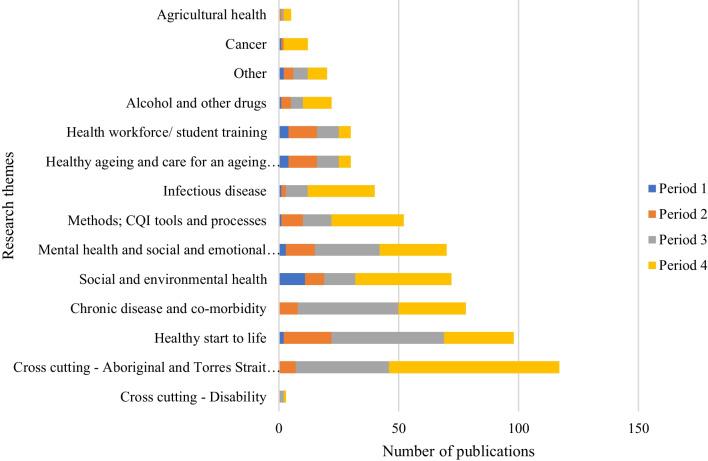
UCRH publications, by research and cross-cutting themes, by periods, from 2002 to 2022

Over the 20-year period, the majority of publications (17%, *n* = 98) focused on the topic of 'healthy start to life'. This was followed by 'chronic disease and co-morbidity' (14%, *n* = 78), 'social and environmental health' (12%, *n* = 72), 'mental health and social and emotional wellbeing' (12%, *n* = 70), and 'methods, and CQI tools and processes' (9%, *n* = 54) (Table 2, Fig. 1).

Over time there was a decrease in the percentage of publications addressing ‘health workforce and student training’ and ‘healthy ageing and care of an ageing population’ (Table 2). On the other hand, the number and percentage of publications related to the research themes of ‘infectious disease’ and ‘methods, CQI tools and processes’ also increased.

In period 1, the majority of publications were focused on ‘social and environmental health’ (37%, *n* = 11). In period 2 and 3, most publications shifted to ‘healthy start to life’ with 18% (*n* = 20) and 25% (*n* = 47) respectively. In period 4, the highest percentage of publications were again related to ‘social and environmental health’ (16%, *n* = 40).

As the centre evolved there was a notable increase in the number of publications related to Aboriginal and Torres Strait Islander health, with 39 publications in period 3 and 71 publications in period 4 (Table 2). However, there was a lack of publications addressing the cross-cutting theme of disability, with only three publications throughout the 20-year period.

### Equity in authorship—Aboriginal and Torres Strait Islander and female authorship

Over time there was an increase in the number and percentage both of Aboriginal and Torres Strait Islander first-authored publications from 0% (*n* = 0) in periods 1–3 to 4% (*n* = 10) in period 4, and of Aboriginal and Torres Strait Islander last-authored publications from 1% (*n* = 2) in period 3 to 4% (*n* = 11) in period 4. This expansion between periods 3 and 4 saw the number and percentage of publications with at least one Aboriginal and Torres Strait Islander author increase from 11% (*n* = 21) to 26% (*n* = 65). Despite this increase, not all publications with an Aboriginal and Torres Strait Islander focus had at least one Aboriginal and Torres Strait Islander author. In period 3, 54% (21/39) of these publications had at least one Aboriginal and Torres Strait Islander author, which increased to 92% (65/71) in period 4.

Female last authors also increased over time, from none in period 1 to 58% (*n* = 143) in period 4. Female first authors increased after the first period to above 70% for the remaining periods.

### Publication metrics

Publications in Quartile 1 Journals increased from 43% (*n* = 13) in period 1 to 64% (*n* = 121) and 63% (154) in periods 3 and 4 respectively (see Table 2). The top three journals were *BMC Health Services, BMJ Open* and *Medical Journal of Australia* (Table 3), all Quartile 1 Journals. Of the 13 different journals with which UCRH-affiliated authors had published 10 or more times, 5 were specifically Australian journals, namely *Australian Health Review, Australian and New Zealand Journal of Public Health, Australian Journal of Primary Health, Australian Journal of Rural Health*, and *Medical Journal of Australia.*

**Table 3:** Peer-review journals with 10 or more publications from UCRH-affiliated authors, 2002–2022

Journals	No. of publications
Australian Health Review	10
Australian and New Zealand Journal of Public Health	12
Women and Birth	13
International Journal of Environmental Research and Public Health	13
BMC Public Health	14
Frontiers in Public Health	15
Australian Journal of Primary Health	18
Midwifery	18
Australian Journal of Rural Health	20
Rural and Remote Health	20
Medical Journal of Australia	21
BMJ Open	22
BMC Health Services Research	24

### Linking people from a variety of organisations

**Fig. 2:**
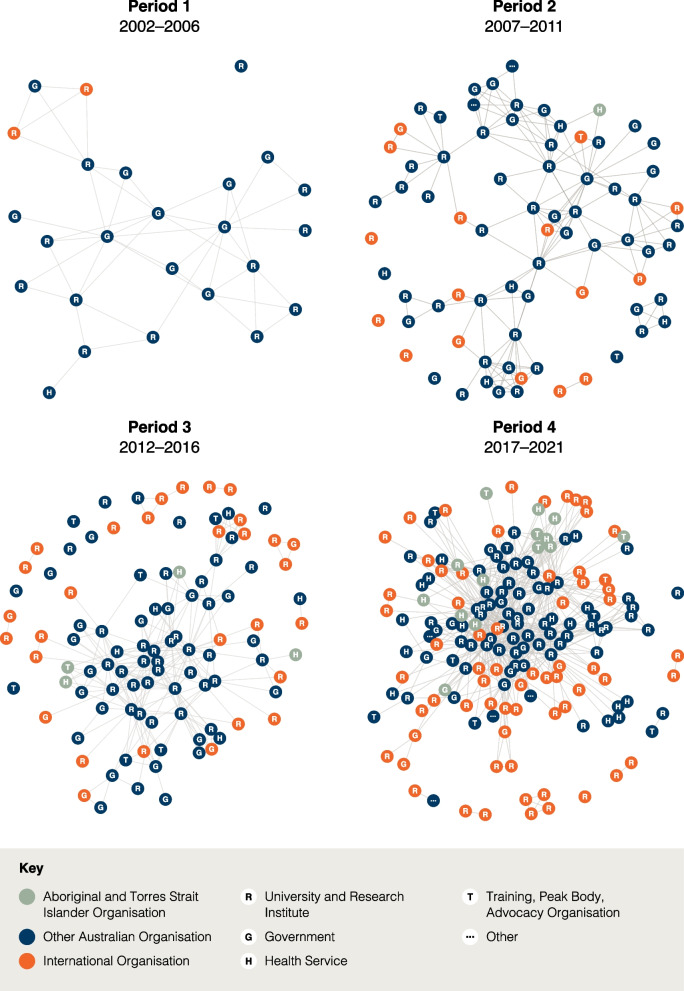
Evolution of the research network, 2002–2021 (using Fruchterman-Reingold layout algorithm)[39]

As shown in Table 4, and the network visualisation in Fig. 2, there was an increase in the number and type of organisations in the network over time, with a considerable growth from period 3 (103 organisations) to period 4 (171 organisations). Of note, the number of universities and research institutes increased from 62 in period 3 to 112 in period 4, while health services rose from 8 to 22. Likewise, engagement with Aboriginal and Torres Strait Islander organisations rose from 4 in period 3 to 14 in period 4 and international organisations rose from 30 to 65.

**Table 4:** Network composition and structural characteristics, by periods

*Main network parameters*	Period of the research network			
	Period 1 2002–2006	Period 2 2007–2011	Period 3 2012–2016	Period 4 2017–2021
Network size (no. of nodes)	25	75	103	171
No. of edges	89	255	472	934
No. and type of organisations				
University or Research Institute	14	41	62	112
Government	10	23	26	21
Health Service	1	6	8	22
Training, Peak Body or Advocacy Organisation	0	3	7	12
Other	0	2	0	4
No. and type of organisations by international, rural, Aboriginal and Torres Strait Islander				
International organisations	2	16	30	65
Rural organisations	4	13	19	23
Aboriginal and Torres Strait Islander organisations	0	1	4	14
Assortativity				
By organisation type	0.080	− 0.023	0.010	0.049
By international organisation	0.288	− 0.022	0.264	0.246
By rural organisation	− 0.077	0.104	0.183	0.373
By Aboriginal and Torres Strait Islander organisation	n.a	n.a	0.110	0.121
Edge distribution				
Minimum degree	0	0	0	0
Median degree	3	3	4	5
Mean degree	3.76	4.4	5.44	8.08
Maximum degree	10	16	25	58
Degree variance	7.02	12.54	28.42	89.06
Network structure				
Network density	0.157	0.059	0.053	0.048
Clustering (average local transitivity)	0.721	0.78	0.725	0.766
Freeman degree centralisation	0.260	0.157	0.192	0.294
No. of separate components	2	9	17	9
No. of isolated nodes	1	6	10	2
Size of the largest component	24	63	78	155
Proportion of nodes in main component	0.96	0.84	0.70	0.84
Network diameter	5	7	6	5

### Relationships of organisations and structural characteristics

The structural characteristics of the network are based on the indicators shown in Table 4 and the visualisation of the network in Fig. 2. We present the findings of the structural characteristics of the network by period to describe the changes over time in a more comprehensive manner.

#### Period 1: 2002–2006

In this period, the UCRH co-authored publications with 25 other organisations, and had 89 co-authoring relationships. Twenty-four of the 25 organisations in the network (96%) are connected within a single network component, with the outlier having only one co-publication with the UCRH but no other research or affiliation with organisations other than UCRH (see the isolate in Fig. 2).

The typical (median) number of co-authoring relationships was three and the maximum was 10. Sixteen per cent of all possible organisational pairs within this network had a co-authoring relationship—the highest value across the four periods. Most organisational co-authors in this network have some other mutual co-authors in common (transitivity is 72%–78% across all periods), partially because of the nature of co-authorship networks in which, by definition, all cliques of authors on the same publication are fully interconnected. Network centralisation—quantifying how much one central organisation can dominate the UCRH co-authoring network—was at 26% of its theoretical maximum.

The diameter of this component is five—i.e., it takes five network steps from the only health service located on one side of this UCRH research network to the two international research organisations on the other side. As these two organisations are connected with each other, the network has a positive international status assortativity score.

#### Period 2: 2007–2011

This period saw a dramatic broadening of the UCRH research network and its reach, with the number of organisations involved tripling to 75. The typical number of co-authoring relationships remained at similar levels as in period 1 (median degree 3) leading to a drop of overall network density of under 6%, i.e., the growth of the network necessitated a large proportion of the institutes involved not having a direct link with one another. The maximum number of collaborations for any institute increased to 16, and the variance in the number of collaboration relationships among different organisations correspondingly increased to almost 13.

However, given the disproportionately faster growth of the network, the structure became less centralised around any single dominant co-authoring organisation, as quantified by the decreasing Freeman degree centralisation score of 0.16 in this larger network. At the same time, there was a widening of the network (to diameter seven) and a fragmentation into nine disconnected components that included six isolated nodes. This meant that UCRH had a higher number of independent pair-wise collaborations with diverse single organisations, none of which had connected through joint publications with others in this period and thereby potentially tapping into diverse pools of knowledge. In addition, more rural (*n* = 13) and international organisations (*n* = 16) from university and research institutes, government and training/advocacy sectors entered various parts of the UCRH research network in this period, mostly as the only international partner on each publication, thus dissolving the previously positive assortativity score. The numbers of Aboriginal and Torres Strait Islander organisations collaborating remained low (n = 4).

#### Period 3: 2012–2016

This period marks the peak of bridging activity and topological diversity of UCRH collaborations. In period 3, the size of the network increased to 103 co-authoring organisations with a higher average number of collaborations (mean 5.44, median 4), which overall produced 472 edges in the network. Despite the higher co-authoring activity, fragmentation of the network continued—the network had 17 disconnected components and 10 isolated nodes—indicating the formation of new channels to diverse and possibly otherwise disconnected organisational domains. The main interconnected component captured the lowest proportion of the network as compared to all other periods (70%)—another measure of the breadth of the overall network and the relatively low redundancy in terms of the spread of relationships in this period. Several of the collaborations were with teams that included multiple international institutes working jointly together (n = 30), resulting in a positive assortativity score of a moderate magnitude (0.264).

#### Period 4: 2017–2021

The final period displays all the hallmarks of network consolidation. Despite the network’s further growth to include 171 collaborating organisations, it became notably more compact. The median number of co-authoring relationships grew to five and the network had 934 edges in total. The University of Sydney emerged as highly dominant in period 4, with 58 co-authoring relationships with the partners of UCRH. This drove the network to the highest levels of Freeman degree centralisation observed across our study despite the network being at its largest during this period.

These high levels of centralisation, coupled with the highest co-authoring activity seen over all four periods, increased the overall network connectivity. While the size of the main interconnected component grew to 155 nodes, its diameter shrank to 5, indicating the same distance from one side of the network to the opposite side as in period 1 when the entire network had only 25 nodes. In addition, UCRH had fewer independent explorations with smaller groups of separate author teams, with only two isolates among the 184 organisations and fewer separate components (*n* = 9). Period 4 also involved the highest number of Aboriginal and Torres Strait Islander organisations (*n* = 14), rural organisations (*n* = 23) and international (*n* = 65) collaborators. While the network positions of the Aboriginal and Torres Strait Islander organisations did not display statistically strong tendencies to cluster together, many of the rural organisations were jointly involved in the same studies as quantified by the positive assortativity score of 0.37.

## Discussion

By assessing organisational co-authorship using both network and descriptive analysis of publications, our study has provided a nuanced understanding of the evolution of an Australian rurally based academic centre over a 20-year period (2001–2021). We identified 577 publications with 130 different UCRH-affiliated authors. Key findings include: (1) expansion in the number of publications and UCRH-affiliated authors; (2) a greater number and diversity of organisations collaborating with the UCRH as reflected in co-authorship; (3) a consolidation of the collaborative network with fewer UCRH authors having fewer independent research endeavours with smaller groups of separate author teams; (4) about two thirds of publications make no reference to regional or remote populations; (5) a notable increase in publications related to Aboriginal and Torres Strait Islander health, coupled with an increase in Aboriginal and Torres Strait Islander authorship; 6) a rise in female senior authorship; and (7) increasing number of publications in high-quality journals.

Research collaboration allows for researchers to draw on a broad range of expertise and perspectives and is necessary for increasing general research productivity [5, 40–42]. In 2015, Gausia et al. [5] observed a wide variation in the publication output from Australian rural academic centres and suggested that collaboration with external organisations enhances research productivity. We found this to be the case in our study, where a dramatic broadening of the network and its reach over time coincided with a sharp increase in the number of organisations co-authoring—from 25 in period 1 to 171 in period 4. Interestingly, however, our findings also suggest that while increasing the number of co-authoring organisations may lead to greater network productivity in terms of publications, it does not necessarily build a more cohesive network. Our results show that as the UCRH grew during Periods 2 and 3, its structure became fragmented and less centralised. This was because co-authoring was predominantly occurring with diverse single organisations that had not connected through joint publications with others in the network. Thus, network growth and consolidation was greatest in Period 4 when there were increases in the number of both publications and collaborating organisations, and fewer independent explorations with smaller groups of separate author teams.

Like Bailey et al. [12], in their examination of a decade of Australian rural health research, we identified a large proportion of publications emanating from the UCRH that were not rural focused. As such, it became evident from our study that building a rural health research network requires collaboration with a variety of organisations, including research organisations in urban and international settings that may not be focused on rural research. Furthermore, this type of inter-organisational partnering has been shown to enhance knowledge creation, and to promote information exchange and the spread of good practice [5, 41–43]. Nevertheless, with the continuing disparities in health between rural and urban populations it is imperative that the work of rural health researchers becomes more focussed on understanding and addressing these disparities in an Australian context.

Research capacity-building opportunities are critical to building and sustaining rural health research [4, 44–46]. In our study we identified an increase in rural research capacity. Over time there was an increase in publications with a UCRH affiliated first or last (senior) author, increase in female last authorship and Aboriginal and Torres Strait Islander authorship, and a rise in publications in quartile 1 journals. Previous studies have found that to achieve this there needs to be targeted investments in collaborations with both internal and external partners [47]. O’Sullivan et al. [4, 45] identified the need to build rural academic pathways to attract more rurally based clinicians and academically trained people already based in rural areas. They also recommended building the rural health research workforce by investing in rurally based Masters and PhD research scholarships, advertising and promoting rural health projects, and building PhD training options within rural-based organisations. In addition to these potential strategies, we propose that there needs to be a vertical investment, not just in building the workforce but also in attracting and retaining senior academics in rural areas.

A positive development identified in this study was that, over time, there was an increase in research on Aboriginal and Torres Strait Islander health, coupled with an increase in authorship by Aboriginal and Torres Strait Islander authors and organisations. Some of this would have been driven by UCRH hosting two NHMRC-funded Centres for Research Excellence—the Centre for Research Excellence in Integrated Quality Improvement (2017–2019) [43, 48] and the Aboriginal and Torres Strait Islander-led Centre for Research Excellence in Strengthening Systems for Indigenous Health Care Equity (2020–2024) [22, 49]. However, there remains much room for improvement in increasing the number of first and last Aboriginal and Torres Strait Islander authorships on publications [24]. This can be achieved through targeted and meaningful investments that will continue to grow the Aboriginal and Torres Strait Islander health research base by ensuring Aboriginal and Torres Strait Islander leadership and participation in research and strengthening ties with First Nations organisations [25, 50].

## Strengths and limitations of the study

One of our study’s strengths was having the long timeframe of 20 years over which to map our publications output. Although it is only one indicator of collaboration, there are several advantages to relying on it as a proxy for assessing the level of research collaboration including its verifiability, its stability over time, the availability of data in the public domain and the ease of measurement [26]. Although the generalisability of the findings may be limited to similar research centres, the methodological approach could readily be transferred.

The limitations of this study include the following: (1) There are many collaborative efforts that are not reflected in publication and co-authorship metrics. Other measures of collaborative ties include having co-investigators on submitted or funded grants, on conference presentations and as authors of grey literature publications. However, we assumed that, in most cases, co-authorship on a publication indicates an active cooperation between partners beyond the simple exchange of material or information. (2) This analysis does not capture the collaborations that continue to occur through co-authorship or other means that are not necessarily related to the research centre. For example, a collaboration formed by co-authoring on a publication might lead to collaborating on other projects and research not reflected in this analysis. (3) Although multiple authorship affiliations are increasingly recognised as facilitating knowledge exchange and becoming more widespread [51], our analysis does not include the multiple affiliations of many of the authors and so may under-report the level of collaboration. Similarly, only representing the university affiliation, and not the actual department in which an author works, obscures collaboration between departments in the same university. (4) Three of the four authors on this paper (JB, MP, RB) had published more than 20 of the manuscripts included in this analysis. Given this, and to mitigate against bias, PM who has not published as part of this network, undertook the network analysis. (5) Our analysis did not examine the types of studies resulting in these publications as this was beyond the scope of this project. A more comprehensive bibliometric analysis of each research theme, examining whether papers were descriptive or reporting interventions, with changes over time, could be explored in future research.

This review is timely as the Australian Government continues to expand its RHMT investment in rurally based academic centres. Drawing on our findings, Box 1 proposes several strategies to further strengthen research networks for rural research centres.

Box 1: strategies to strengthen research networks for rural research centres

- Targeted mentoring and support for rurally based researchers to link with established research teams.
- Support for development of research collaborations to specifically address rural health inequities and rural–urban disparities in health.
- Vertical investment in building rural academic pathways including:

- Targeted scholarships for rural students undertaking research degrees.
- Funding to attract and retain senior researchers with a track record of success in competitive grants.
- Targeted competitive funding for research addressing rural health inequities.

- Employing and strengthening the capacity of Aboriginal and Torres Strait Islander researchers, including through the above mechanisms.

## Conclusions

Assessing organisational coauthorship using both network and descriptive approaches has been useful for demonstrating the evolution of an Australian rural research centre. Over the 20-year time frame, numbers of publications increased as collaboration in publications increased, expanded, and consolidated, particularly in the final period. This coincided with an increase in the number and diversity of both co-authoring organisations and UCRH-affiliated authors. The findings highlight the value of collaboration in building rural health research capacity. With increased capacity and consolidation of the network it is now imperative that the collaborative research becomes more focussed on understanding and addressing rural health inequities and strengthening the involvement of Aboriginal and Torres Strait Islander people in rural research. This review is timely as the Australian Government continues to expand its investment in rurally based academic centres.

## Data Availability

Data are available on reasonable request. The data set is available from the corresponding author on reasonable request.

## Declarations

### Patient and public involvement

Patients and the public were not involved in the design, or conduct, or reporting, or dissemination plans of this research.
